# Supplementary material for: Lactiplantibacillus plantarum from Unexplored Tunisian Ecological Niches: Antimicrobial Potential, Probiotic and Food Applications
Source: Microorganisms. 2023 Oct 31;11(11):2679. doi: 10.3390/microorganisms11112679 (PMC10673251; doi:10.3390/microorganisms11112679)
Supplement: Supplementary file 1 [file microorganisms-11-02679-s001.zip › microorganisms-2683232-supplementary.pdf]

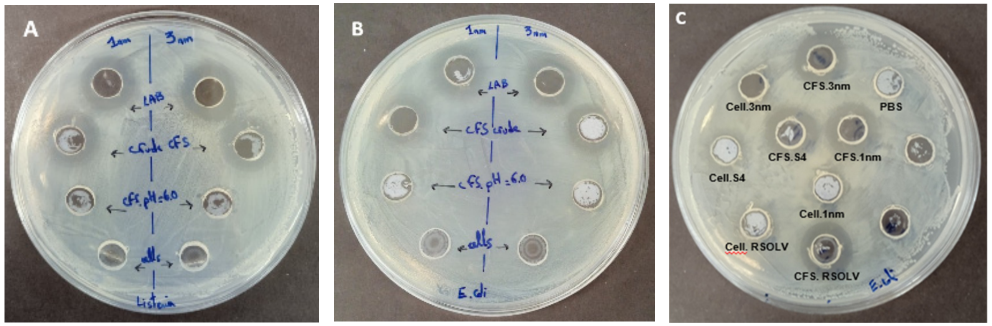

FigureS1. Antibacterial activity of some *Lpb. plantarum* strains and their CFSs (crude CFS, neutralized CFS pH=6.0, cells and heat treated cells) against *L. monocytogenes* (A) and *E.coli* (B and C).

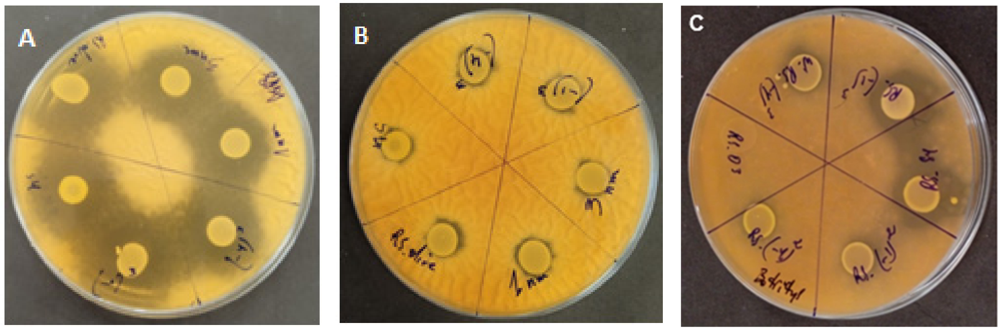

FigureS2. Antifungal activity of *Lpb. plantarum* strains against some indicator fungi species: *Penicillium expansum* (A), *F. culmorum* CECT 2148 (B) and *Botrytis cinerea* CECT 20973 (C)

Table S1. Pathogen antagonism by *Lpb. plantarum* on tomatoes. CFU counts of *Lpb. plantarum* inoculated in MRS as pure culture and inoculated as co-culture with pathogen (*L. monocytogenes* CECT 4031 or *E.coli* O157:H7) . Mean ± standard deviation from three independent CFU counts from three different experiments. Each experiment was done in duplicate. Log CFU per tomato compared to single inoculated pathogen used as control. Two-

way ANOVA, Dunnett's multiple comparisons test are used to compare strains at different time. [§] *p*<0.05 *vs* 5 days 3nm-*Listeria*, 1nm-*Listeria*, RSOLV-*Listeria* and pepp1-*Listeria*; [&] *p*<0.05 *vs* 5 days pepp2-*Listeria*.

	**Number of tomato-attached *Lpb. plantarum* (Log CFU/g in MRS)**			
	**Medium agar**	**1 day**	**3 days**	**5 days**
***E.coli* O157:H7-3nm**	MRS	1.83±1.26	1.78±1.53	1.85±1.31
***E.coli* O157:H7-1nm**	MRS	1.84±1.36	1.60±0.54	1.60±1.34
***E.coli* O157:H7-S4**	MRS	1.86±1.28	1.54±0.51	1.73±1.26
***E.coli* O157:H7-RSOLV**	MRS	1.82±1.05	1.60±0.69	1.72±1.20
***E.coli* O157:H7-pepp1**	MRS	1.93±1.36	1.52±0.98	1.65±0.39
***E.coli* O157:H7-pepp2**	MRS	1.91±1.06	1.42±1.26	1.59±1.18
***L. monocytogenes* CECT 4031 -3nm**	MRS	1.86±1.16	1.75±1.18	1.90±0.94
***L. monocytogenes* CECT 4031 -1nm**	MRS	1.83±0.63	1.36±0.73	1.59±0.40
***L. monocytogenes* CECT 4031 –S4**	MRS	1.72±0.55	1.75±0.67	1.04±0.15[§]
***L. monocytogenes* CECT 4031 -RSOLV**	MRS	1.70±0.80	1.74±1.29	1.85±0.43
***L. monocytogenes* CECT 4031 –pepp1**	MRS	1.93±0.88	1.57±1.46	2.00±1.44[&]
***L. monocytogenes* CECT 4031 –pepp2**	MRS	1.98±1.60	1.61±1.20	1.63±0.84
**3nm (control)**	MRS	1.80±1.30	1.59±1.20	1.68±1.09
**1nm (control)**	MRS	1.80±1.10	1.40±0.33	2.15±0.55
**S4 (control)**	MRS	1.66±1.56	1.81±0.60	1.79±0.93

**RSOLV (control)**	MRS	1.93±1.77	1.14±1.29	1.10±0.55
**pepp1 (control)**	MRS	1.87±1.15	1.43±0.45	1.75±0.03
**pepp2 (control)**	MRS	1.82±1.67	1.01±0.45	1.02±0.15
